# Combined magnetic resonance coronary artery imaging, myocardial perfusion and late gadolinium enhancement in patients with suspected coronary artery disease

**DOI:** 10.1186/1532-429X-10-45

**Published:** 2008-10-17

**Authors:** Christoph Klein, Rolf Gebker, Thomas Kokocinski, Stephan Dreysse, Bernhard Schnackenburg, Eckart Fleck, Eike Nagel

**Affiliations:** 1 German Heart Institute Berlin, Germany; 2 Philips Medical Systems, Hamburg, Germany; 3 Kings College, London, UK

## Abstract

**Background:**

Cardiovascular Magnetic Resonance (CMR) imaging offers methods for the detection of ischemia and myocardial infarction as well as visualization of the coronary arteries (MRCA). However, a direct comparison of adenosine perfusion (PERF), late gadolinium enhancement (LGE) and MRCA or the results of their combination has not been performed. Aim of the study was to evaluate the feasibility/diagnostic performance of rest/stress perfusion, late gadolinium enhancement and MRCA and their combination in patients with suspected coronary artery disease (CAD) in comparison to invasive angiography.

**Methods:**

Fifty-four patients (60 ± 10 years, 35 men, CAD 48%) underwent CMR including MRCA (steady state free precession, navigator whole heart approach, spatial resolution 0.7 × 0.7 × .0.9 mm, trigger delay and temporal resolution adjusted individually), stress PERF (adenosine 140 μg/min/kg), rest PERF (SSFP, 3 short axis, 1 saturation prepulse per slice) and LGE (3D inversion recovery technique) using Gd-BOPTA. Images were analyzed visually. Stenosis >50% in invasive angiography was considered significant.

**Results:**

Mean study time was 68 ± 11 minutes. Sensitivity for PERF, LGE, MRCA and the combination of PERF/LGE and PERF/LGE/MRCA was 87%, 50%, 91%, 88% and 92%, respectively and specificity 88%, 96%, 46%, 88% and 56%, respectively. If image quality of MRCA was excellent (n = 18) the combination of MRCA/PERF/LGE yield a sensitivity of 86% and specificity of 91%. However, no test or combination improved diagnostic performance significantly compared to PERF alone.

**Conclusion:**

In patients with CAD, the combination of stress PERF, LGE and MRCA is feasible. When compared to invasive angiography, adenosine stress perfusion outperforms CMR coronary angiography in direct comparison and yields the best results with non-significant improvement in combination with LGE and significant deterioration in combination with MRCA. MRCA may be of additional value only in a minority of patients with excellent image quality.

## Background

Cardiovascular magnetic resonance (CMR) has emerged as a useful clinical tool for the detection and characterization of coronary artery disease (CAD). It offers functional studies for the detection of ischemia, tissue characterisation for the detection and quantification of myocardial infarction as well as luminal assessment of the coronary arteries. Several single and one multicenter trial have demonstrated high diagnostic accuracy of adenosine perfusion imaging [1-7] with potential advantages (e.g. higher spatial resolution) compared to nuclear imaging [8]. Infarct imaging has proven to be in concordance with histology [9] and more sensitive compared to nuclear imaging [10,11], as small subendocardial defects can be detected. In patients without previous history of CAD the combination of perfusion and infarct imaging can increase diagnostic accuracy compared to perfusion alone, especially by increasing specificity [2,12] in cases with suboptimal perfusion image quality (IQ). A recent meta-analysis of published data on CMR coronary angiography (MRCA) demonstrated a sensitivity and specificity of 88% and 56%, respectively[13]. The only multicenter trial confirmed a high sensitivity and low specificity for the detection of CAD [14]. More recent single center CMR-coronary angiographic data demonstrated superior results, especially improved specificity [15,16]. Aim of the present study was to assess the feasibility and diagnostic accuracy of CMR stress/rest adenosine perfusion, infarct imaging and coronary angiography and their combination for the detection of significant stenosis in patients with suspected CAD scheduled for invasive coronary angiography.

## Methods

The prospective study was approved by the institutional review board of the Charité, Berlin, Germany. Fifty-five consecutive patients with suspected CAD who were referred for invasive coronary angiography were prospectively included into the study after given informed consent. Patients with contraindications for CMR, known myocardial infarction, atrial fibrillation, instable angina, AV block > I°, obstructive lung disease or claustrophobia were excluded from the study.

### Magnetic resonance imaging

**Figure 1:**
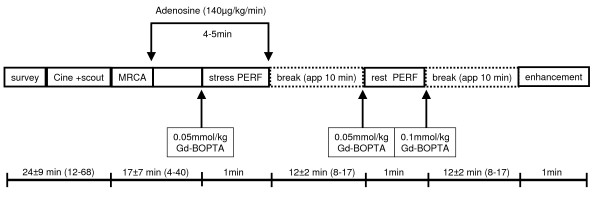
**Study protocol and duration of the different examinations.** Cine + scout includes the coroscout, left ventricular function and the determination of beginning and duration of the cardiac resting period.

All patients were examined in supine position using a 1.5 Tesla scanner (Intera, Philips Medical Systems, Netherlands). A five-element cardiac synergy coil was used for signal detection. The study protocol is shown in Figure 1. The 4-chamber view was imaged with 40 phases/cardiac cycle to visually determine the timing and duration of the individual cardiac rest period [17]. To adequately visualize the most cranial and caudal dimension of the coronary system, a coronary whole heart scout in transversal orientation was used. A sufficient number of strictly transversal slices (120–140) were then obtained to cover the whole heart (steady state free precession (SSFP)), fat suppression, T2 preparation pre-pulse; SENSE factor 1.7; TR/TE/FA 4.6/2.3/100°; trigger delay and temporal resolution adjusted to individual diastolic coronary rest period). Spatial resolution was nearly isotropic (0.7 × 0.7 × 0.9 mm^3^). Breathing motion was compensated using a cranio-caudal navigator technique. The gating window was set to 6 mm. First pass stress perfusion (PERF) (SSFP, TE/TR/FA 2.7/1.4/50°, 1 saturation prepulse per slice, 3 short axis slices/heart beat) was begun after 3 minutes of i.v. adenosine infusion (140 μg/min/kg body weight) and a peripheral bolus of 0.05 mmol/kg body weight Gd-BOPTA (MultiHance^©^, Altana, Germany). After a period of app. 10 minutes to allow for clearance of the contrast agent, rest perfusion (0.05 mmol/kg Gd-BOPTA) was performed, followed by additional 0.1 mmol/kg Gd-BOPTA. Late gadolinium enhancement (LGE) was imaged in short axis and the standard long axis views after 10 minutes using an inversion recovery 3D-turbo-gradient-echo-technique (TE/TR/FA 2.3/4.8/15°, spatial resolution 1.4 × 1.4 × 5.0 mm^3^, acquisition time 215 ms, prepulse delay 225 – 300 ms).

### Image Analysis

All CMR images were evaluated visually on the commercially available ViewForum (Philips, Best, Netherlands) using the 16 segment model by agreement of two experienced (>5 years of CMR) observers fully blinded to the results of the invasive coronary angiography and the other CMR exams. For the combination of tests a patient was classified as having CAD if any of the tests was positive.

#### Perfusion and late Gadolinium enhancement

For the perfusion analysis the adenosine perfusion images were compared side by side with the rest perfusion (e.g. artifacts). In a second seperate turn the perfusion images were analysed in combination with the enhancement images. The presence and transmural extent of a perfusion defect were determined in the dynamic image with the maximal extent of the defect. A perfusion defect was graded visually as subendocardial (<75%) or transmural (≥ 75%). Any regional stress induced defect or late Gadolinium enhancement in any segment was considered positive.

#### CMR coronary angiography

For the visual assessment of coronary artery stenoses, the unprocessed raw data were used. The diagnostic performance was determined in a 16-segment model: (i) left main segment, (ii) proximal, (iii) mid and (iv) distal segment of LAD, (v) first and (vi) second diagonal branch, (vii) proximal, (viii) mid and (ix) distal segment of LCX, (x) first and (xi) second marginal branch, (xii) proximal, (xiii) mid and (xiv) distal segment of RCA, (xv) right posterolateralis segment, and (xvi) posterolateral descending artery segment.

Image quality of the entire 3D-data set was visually graded as excellent (coronary artery visible with sharply defined borders), good (mildly blurred borders), moderate (moderately blurred borders) or non-diagnostic (markedly blurred borders) [14]. The latter were not included into the analysis. Patients were classified as having or not having CAD. For the final results only vessels with a diameter suitable for revascularization (≥ 2 mm, visual assessment) in invasive angiography were included.

### Invasive coronary angiography

All coronary X-ray angiographies were performed within 24 hours after CMR examination. Two experienced interventional cardiologist blinded to the results of the CMR examinations visually evaluated the angiograms. A hemodynamically significant coronary stenosis was defined as >50% luminal diameter narrowing.

### Statistics

Statistical analysis was performed using SPSS 12.0.1 for Windows (SPSS Inc.). For all continuous parameters mean ± SD are given. Sensitivity, specificity, and diagnostic accuracy including the confidence intervals were calculated according to standard definitions. For comparison between the tests a nonparametric test (McNemar) was used. Values <0.05 were considered significant. For the combined interpretation, patients with a non-diagnostic perfusion scan, but pathological LGE or MRCA were included, those with a non-diagnostic perfusion scan and a negative LGE or MRCA excluded from the analysis.

## Results

The patients characteristics are shown in Table 1. Of the 54 patients 26 (48%) had significant CAD (12 one-vessel, 8 two-vessel and 6 three-vessel disease). Mean study time (patient in the scanner) was 68 ± 11 minutes (range 52–118 min) mainly due to different durations of data acquisition of MRCA. Study times of the different modules are shown in Figure 1. Table 2 demonstrates the sensitivities, specificities and diagnostic accuracy.

**Table 1:** Patient characteristics

	**Entire group (n = 54)**	**CAD (n = 26)**	**No CAD (n = 28)**	**p**
**Male/female**	35/19	22/4	13/15	**0.004**

**Age (years)**	60 ± 10 (37–78)	60 ± 9 (41–77)	60 ± 11 (37–78)	0.73

**Weight (kg)**	81 ± 15 (54–118)	86 ± 15 (54–118)	76 ± 14 (55–104)	**0.03**

**BMI**	27.6 ± 4.1 (21.1–36.7)	28.4 ± 4.2 (21.1–36.7)	26.8 ± 3.9 (21.5–34.9)	0.10

**Typical angina**	30 (56%)	20 (77%)	10 (36%)	**0.003**

**Atypical angina**	15 (28%)	4 (15%)	11 (39%)	0.05

**Dyspnoea on exertion**	21 (39%)	6 (23%)	15 (54%)	**0.02**

**DM**	12 (22%)	5 (19%)	7 (25%)	0.61

**Hypertension**	37 (69%)	22 (85%)	15 (54%)	**0.02**

**Smoker**	18 (33%)	11 (42%)	7 (25%)	0.18

**Hypercholesterinemia**	41 (76%)	21 (81%)	20 (71%)	0.43

**Family history**	17 (31%)	9 (35%)	8 (29%)	0.64

**Pathological ECG**	16 (30%)	9 (35%)	7 (25%)	0.68

**LV ejection fraction**	59 ± 9% (31–71)	57 ± 10% (31–67)	61 ± 7% (39–71)	0.18

**Positive nuclear study**	13 (24%)	6 (23%)	7 (25%)	

**Positive stress echo**	5 (9%)	1 (4%)	4 (14%)	

**Positive exercise test**	8 (15%)	6 (23%)	2 (7%)	

CAD = coronary artery disease; BMI = body mass index; DM = Diabetes mellitus; ECG = electrocardiography; LV = left ventricular.

**Table 2:** Diagnostic accuracy of the individual test and their combination on a patient basis

	**Sensitivity (%) (95% CI)**	**Specificity (%) (95% CI)**	**Accuracy (%) (95% CI)**
**PERF **(n = 49)	**87 (65;97) **[20/23]	**88 (69;97) **[23/26)]	**88 (75;95) **[43/49]

**LGE **(n = 54)	**50 (30;70) **[13/26]	**96 (80;100) **[27/28]	**74 (60;85) **[40/54]

**MRCA **(n = 46)	**91 (69;98) **[20/22]	**54 (33;74) **[13/24]	**70 (54;82) **[32/46]

**PERF/LGE **(n = 51)	**88 (68;97) **[22/25]	**88 (69;97) **[23/26]	**88 (75;95) **[45/51]

**PERF/LGE/MRCA **(n = 51)	**92 (73;99) **[24/26]	**60 (39;78) **[15/25]	**75 (60;80) **[38/51]

PERF = adenosine stress perfusion, LGE = late gadolinium enhancement, MRCA = magnetic resonance coronary angiography.

### Perfusion/late gadolinium enhancement

PERF was not performed in 3 (6%) patients, due to possible aortic stenosis unknown prior to the CMR-exam (1) or severe dyspnoea during adenosine (2). Analysis could not be performed in 2 (4%) patients due to non diagnostic image quality (IQ) due to breathing (1) and trigger artefacts (1). Heart rate increased (p < 0.001) from 72 ± 13/min (52–109/min) to 88 ± 14/min (54–127/min) with adenosine, blood pressure from 131 ± 19/73 ± 12 mmHg to 133 ± 21/73 ± 13 mmHg (p > 0.05). Over all and vessel specific diagnostic performance is shown in Table 2. Of the 10 patients with a transmural perfusion defect, 6 had subendocardial and 3 transmural LGE.

Two of 3 false positive readings were in the RCA territory. One patient had a regional wall motion abnormality, subendocardial enhancement and a perfusion defect at the same site, however, without high grade coronary stenosis, probably due to a small infarction without remaining stenosis.

Of the 3 patients with false negative readings 2 had one-vessel-disease (distal LAD and first diagonal branch), the other three-vessel-disease with 50% left main and 90% medial RCA. Sensitivity of the 14 patients with proximal CAD was 93% (13/14). Enhancement could be analysed in all patients. Fifteen patients showed some degree of enhancement.

### MRCA

Scan duration depended on heart rate (73 ± 15 bpm, range 54–115 bpm) and navigator efficiency (52 ± 13%, range 16–79%) and lasted 6'15" ± 1'36" (range 3'57" – 12'4"). In 7 patients MRCA had to be restarted due to diaphragmatic drift. Images could be acquired in all patients, however, 8 patients (15%) yielded non-diagnostic IQ. Of the remaining, 18 (33%) had excellent, 17 (31%) good and 11 (20%) moderate IQ. Of the 714 segments 521 had a diameter ≥ 2 mm visually defined by invasive angiography, of which 404 (78%) were visualized by CMR (100% of proximal, 87% of medial and 65% of distal segments, 42% of diagonal, 40% of marginal and 54% of RCA side branches). Diagnostic accuracy is shown in Tables 2 and 3. MRCA was significantly inferior (p = 0.002) to PERF. Sensitivity and specificity on a segmental basis was 74% (32 of 43) and 89% (323 of 361), respectively. If IQ is taken into account sensitivity, specificity and diagnostic accuracy on a patient basis were 86%, 91% and 88% for excellent image quality, 100%, 22% and 61% for good image quality and 86%, 25% and 64% for moderate image quality, respectively (Table 4). Body weight and BMI of patients with excellent IQ was 71 ± 11 kg and 25.6 ± 3.4 kg/m^2^, compared to 85 ± 15 kg and 28.4 ± 4.0 kg/m^2 ^in the remaining patients.

**Table 3:** Sensitivity and specificity in percent (%) including the 95% confidence interval of the individual test and their combination on a coronary artery basis

	**LAD**		**LCX**		**RCA**	
	**Sens**	**Spec**	**Sens**	**Spec**	**Sens**	**Spec**

**PERF ** (n = 49)	**86 (56;97) ** [12/14]	**97 (83;100) ** [34/35]	**73 (39;93) ** [8/11]	**89 (74;97) ** [34/38]	**75 (43;93) ** [9/12]	**92 (77;98) ** [34/37]

**LGE ** (n = 54)	**31 (12;59) ** [5/16]	**100 (89;100) ** [38/38]	**38 (15;68) ** [5/13]	**100 (89;100) ** [41/41]	**50 (22;78) ** [6/12]	**95 (83;99) ** [40/42]

**MRCA ** (n = 46)	**100 (72;100) ** [13/13]	**64 (45;79) ** [21/33]	**75 (43;93) ** [9/12]	**85 (68;94) ** [29/34]	**82 (48;97) ** [9/11]	**83 (66;93) ** [29/35]

**PERF/LGE ** (n = 51)	**87 (58;98) ** [13/15]	**94 (80;99) ** [34/36]	**69 (39;90) ** [9/13]	**89 (74;97) ** [34/38]	**75 (43;93) ** [9/12]	**85 (69;94) ** [33/39]

**PERF/LGE/MRCA ** (n = 51)	**100 (76;100) ** [16/16]	**60 (42;76) ** [21/35]	**85 (54;97) ** [11/13]	**75 (57;87) ** [27/36]	**100 (70;100) ** [12/12]	**72 (55;85) ** [26/36]

LAD = left anterior descending, LCX = left circumflex, RCA = right coronary artery, Sens = sensitivity, Spec = specificity, PERF = adenosine stress perfusion, LGE = late gadolinium enhancement, MRCA = magnetic resonance coronary angiography.

**Table 4:** Diagnostic accuracy in patients with excellent and excellent/good image quality in MRCA

	**Sensitivity (%) (95% CI)**	**Specificity (%) (95% CI)**
**PERF/LGE **(IQ in MRCA excellent)	**71 (30;95) **[5/7]	**100 (68;100) **[11/11]

**MRCA **(§) (IQ in MRCA excellent)	**86 (42;99) **[6/7]	**91 (57;100) **[10/11]

**PERF/LGE/MRCA **(§) (IQ in MRCA excellent)	**86 (42;99) **[6/7]	**91 (57;100) **[10/11]

**PERF/LGE **(IQ in MRCA excellent or good)	**87 (58;98) **[13/15]	**90 (68;98) **[19/21]

**MRCA **(*) (IQ in MRCA excellent or good)	**93 (66 ;100) **[14/15]	**57 (34 ;77) **[12/21]

**PERF/LGE/MRCA **(*) (IQ in MRCA excellent or good)	**93 (66;100) **[14/15]	**57 (34;77) **[12/21]

IQ = image quality, PERF = adenosine stress perfusion, LGE = late gadolinium enhancement, MRCA = magnetic resonance coronary angiography. (*) = p < 0.05 compared to PERF/LGE, (§) = p > 0.05 compared to PERF/LGE

### Combination

The sensitivities and specificities shown in Tables 2 and 3 were defined if any of the tests – perfusion, enhancement or MRCA – was positive. If LGE was added to the CMR exam, sensitivity increased (p > 0.05) because two patients without PERF (not performed or non-diagnostic) showed enhancement. Adding LGE did not change the results, if only patients, in whom PERF and LGE were diagnostic were analyzed. If MRCA is added, sensitivity further increased, however specificity decreased (p = 0.001) due to the rate of false positive readings. Subgroup analysis for patients with excellent or both, excellent and good MRCA IQ are shown in Table 4. The combined tests in patients with excellent IQ did not, in patients with excellent and good IQ did differ statistically significant (p = 0.02). In the five patients without PERF 2 had non diagnostic IQ in MRCA, 2 were correct positive and one was false positive.

## Discussion

The present study demonstrates the feasibility of the combination of perfusion, infarct and coronary artery imaging in patients with suspected CAD. Perfusion imaging by itself is the most accurate test. The accuracy can not be significantly improved by adding late gadolinium enhancement. CMR coronary angiography is significantly inferior to PERF and its addition to PERF/LGE decreases diagnostic accuracy. Even if MRCA image quality is excellent it does not show an additional benefit in predicting luminal stenoses.

As invasive coronary angiography includes disadvantages as invasiveness, exposure to radiation, potential life threatening risk, limited information about coronary hemodynamics and high cost, a non invasive modality with the potential to detect and localize ischemia and/or infarction with additional information on coronary anatomy, location and severity of coronary lesions could better select patients to undergo an invasive procedure. CMR can offer a combined protocol in little more than 1 hour with high patient acceptance (all patients in the current study). Potentially, total scan time could be reduced to less than one hour by performing the MRCA between the two perfusion scans [18].

### Perfusion/late gadolinium enhancement

First pass perfusion is the most widely used CMR-technique for the detection of reduced myocardial blood flow and yields superior results compared to SPECT [8]. Our results (sensitivity/specificity 87%/88%) are similar to published data with sensitivities of 84%–93% and specificities of 58%–85% [1-3,6,19]. Proximal CAD was detected in all but one patient. This patient with significant left main (50%) and RCA stenosis, however, did not demonstrate an adenosine induced change in heart rate, blood pressure or of the myocardial perfusion reserve index (upslope) in any of the segments, even if the epicardium was measured separately (data not shown). Although the patient denied any nicotine, caffeine or nitrate consumption 24 h prior to the examination, the lack of any adenosine effect may be the main reason for the false negative result. In general, first pass perfusion in patients with three vessel disease has a high sensitivity. This is supported by the fact that all but one patient with a transmural stress perfusion defect also had enhancement at this site, while patients even with high grade stenosis, but without enhancement had subendocardial stress induced defects only. This demonstrates the ability of the myocardium to preserve perfusion in the epicardium during adenosine vasodilatation even in high grade stenosis. It therefore seems unlikely that 3-vessel-disease has a globally (endo- and epicardial) reduced perfusion reserve that remains undetected by CMR, although the ability of the exact vessel specific localization of ischemia in multi-vessel disease may be reduced [12]. The other two false negative results were in patients with distal and side branch CAD.

LGE is an excellent technique for the detection and quantification of myocardial infarction [9,20]. Whereas a positive finding in a patient without previously known CAD has a strong indication for coronary angiography, its value to characterize patients for myocardial ischemia is limited.

### MRCA

Although using SSFP with a better signal and contrast to noise ratio in comparison to previous T2 prepared turbo gradient techniques [21,22] our approach resulted in a low specificity. In addition15% of patients had non-diagnostic image quality, 35% of distal segments and 60% of side branches were non-diagnostic. Therefore, the current implementation of MRCA is still not sufficiently reliable for the diagnosis and characterisation of CAD. Only in patients with excellent IQ (33%) an acceptable diagnostic accuracy was achieved. Even in patients with good IQ (31%) specificity already dropped considerably, apparently as MRCA defects can either represent true lesions or artefacts. However, due to the small number these results have to be interpreted with care and need to be confirmed in a larger patient group. Our results remain inferior to recently published data [15,16]. This may be due to several reasons. Jahnke et al used a sophisticated motion adaptation technique (affine transformation) not available at the time of the study and also not available for most sites performing CMR studies. In this study analysis was performed on a segmental basis, which yields a higher specificity due to the high number of normal segments. In addition, the whole image procedure was optimized on coronary artery imaging only, allowing up to 30 minutes just for MRCA imaging (without using parallel imaging). However, segmental comparison between CMR and invasive angiography bears the problem of anatomical correlation as segmental definition depends on the identification of side branches that may be different within the two methods, especially as side branches in CMR may easily be missed (more than 50% in our study). Sakuma et al. achieved superior image quality and specificity in 131 Japanese patients with suspected CAD using a similar MRCA protocol. Two major differences to our approach may explain this superior accuracy. First, their patient population was much slimmer (63.4 ± 10.3 kg) compared to ours (81 ± 15 kg). Interestingly, our subgroup of patients with excellent IQ was significantly thinner (71 ± 11 kg), than the overall group. A number of factors (e.g. breathing patterns, heart rate, heart rate variability, scan time, etc.) may influence image quality of free-breathing MRCA. Sakuma et al supplied nitrates before MRCA, which, however, would have influenced our perfusion results. We believe that the current strength or CMR lies in the assessment of hemodynamic consequences of coronary lesions with a growing contribution of vascular morphology. We would, thus, rather integrate MRCA into ischemia testing than improve MRCA on the cost of not performing stress testing. This is of special importance, as it was recently demonstrated that CMR stress testing [23,24] and the detection of scar [25] has a prognostic value in patients with suspected CAD. Potential improvement in MRCA may be achieved with the use of higher field strength (e.g. 3T) [26] or the use of intravascular contrast agents [27,28].

### Combination of tests

**Figure 2:**
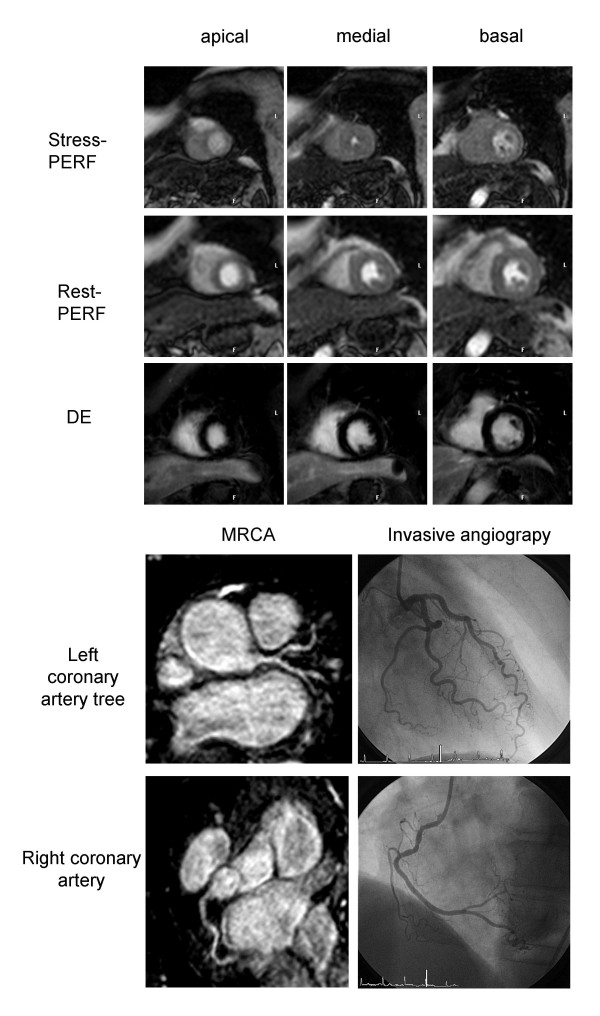
**Example of one patient without significant CAD. **Normal findings in stress- and rest-PERF (short axis views), late gadolinium enhancement (short axis view) and magnetic resonance coronary angiography (SoapBubble software, Philips Medical Systems, Best, the Netherlands). Invasive angiography demonstrates normal coronary arteries.

**Figure 3:**
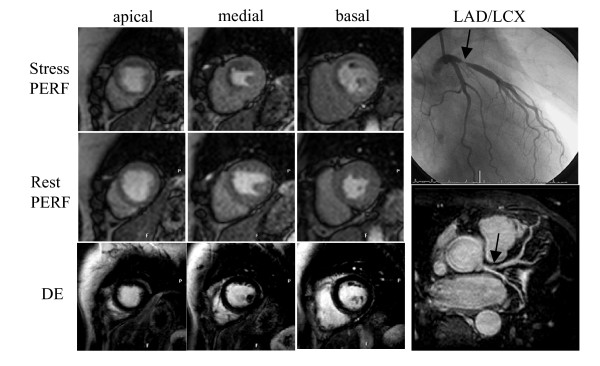
**CMR study of a patient with a high grade (90%) left anterior descending artery (LAD) stenosis (arrow). **There is a large regional perfusion defect in the LAD territory, including all segments of the apical slice and the septal and anterior segments of the medial and basal slices (short axis views). No late gadolinium enhancement was detected. Although a clear defect in the proximal LAD can be appreciated in the MRCA (SoapBubble software, Philips Medical Systems, Best, the Netherlands), the high grade filiform stenosis is not imaged appropriately.

Figures 2 and 3 show examples of a patient without and with significant CAD. The best result was achieved with the combination of stress perfusion and late gadolinium enhancement (not significant). However, the combination was only superior to perfusion alone, as perfusion was non-diagnostic in two patients with a positive late gadolinium enhancement. Improved sensitivity can be achieved in patients with chronic infarction without a stress induced perfusion defect. Myocardial scar, however, has a low perfusion at rest with hardly any perfusion reserve [29] and therefore, the defect should be more pronounced during stress. Small subendocardial infarcts may be detected by LGE due to the high spatial resolution in comparison to perfusion, which, however, was not the case in our patient population. Although not strongly supported by our study, we support the use of LGE when a perfusion study is performed, as the presence of scar is an important information, not necessary drawn from perfusion alone and if present adding confidence to diagnose CAD. The addition of MRCA did not improve accuracy. The main issue remains the difficulty to achieve adequate image quality within the combined approach. With the current techniques, the role of MRCA remains limited, except to gain further confidence if excellent image quality can be obtained. This may be of specific importance in patients with microvascular disease who may have a positive perfusion scan, but no treatable epicardial stenosis. Our results, however, are different to a recent report by Plein et al. [30] who demonstrated a small, but additional value of MRCA compared to perfusion alone. Possible explanations could be first the very different patient population with non-ST-segment elevation acute coronary syndrome and a high prevalence of significant CAD. Second, Plein et al used a targeted compared to a whole heart approach and third, the authors analysed the proximal and medial portions of the left and circumflex coronary only and were allowed to discard images with poor IQ or significant artefacts, thus, possibly reducing false positive readings.

### Patient population

There was an intermediate pre-test probability for CAD (48%) with similar cardiovascular risk factors in the groups with and without CAD (except hypertension). In both groups there were individuals with typical and atypical angina, dyspnoea on exertion and ECG changes, therefore making a non-invasive test desirable. Care was taken to include patients prospectively without exclusion of unfavourable patients like high BMIs or diabetes (Table 1).

### Limitations

There are, however, certain limitations to the study. Due to the rapid development, especially of MRCA, there are techniques available that may outperform the method used in our study. The technique applied, however, is commercially available to many cardiovascular CMR users without research tools. Therefore, this technique needs to be evaluated for its clinical use in comparison with functional tests. The patient number is relatively low. We are, however, confident that even in a larger patient population, the results of the limited advantage of MRCA would not have changed as only 33% of patients achieved image quality that may be of additional value. Our patients were referred for invasive coronary angiography, therefore representing a highly selected patient population. Our results can therefore not be transferred to a more unselected group of patients. And last, we have compared a functional imaging test (PERF) with the morphology of the coronary arteries as the gold standard without the addition of additional functional assessment. Therefore we cannot be absolutely certain about the "real" functional relevance of a stenosis. However, although not optimal, the majority of studies assessing non-invasive testing have compared their results to angiography.

## Conclusion

In conclusion, the combination of functional and morphological studies with CMR in one session is feasible. Adenosine perfusion is a robust technique in patients with suspected CAD and outperforms CMR coronary angiography. It should be combined with late Gadolinium enhancement, however, an additional benefit of CMR coronary imaging may only be demonstrated in patients with excellent image quality.

## Abbreviations

BMI: Body mass index; CAD: Coronary artery disease; CMR: Cardiovascular magnetic resonance; FA: Flip angle; IQ: Image quality; LAD: Left anterior descending coronary artery; LCX: Left circumflex coronary artery; LGE: Late gadolinium enhancement; MRCA: Magnetic resonance coronary angiography; PERF: First pass perfusion; RCA: Right coronary artery; SSFP: Steady state free precession; TE: Echo time; TR: Repetition time.

## Competing interests

Bernhard Schnackenburg is employee of Philips Medical Systems, Hamburg, Germany.

## Authors' contributions

CK designed and coordinated the study, performed CMR image analysis and drafted the manuscript. RG performed CMR image analysis and carried out CMR-exams. TK performed CMR image analysis and carried out CMR-exams. SD performed image analysis (invasive angiography). BS conceived of the study and participated with the design of the study. EF performed image analysis (invasive angiography) and participated in the design of the study. EN conceived of the study, participated in its design and helped with the revision of the manuscript. All authors have revised the manuscript and have read and approved the final version.
